# Supplementary material for: A methodology for the design of experiments in computational intelligence with multiple regression models
Source: PeerJ. 2016 Dec 1;4:e2721. doi: 10.7717/peerj.2721 (PMC5136129; doi:10.7717/peerj.2721)
Supplement: Data S1 — Datailed results from UC Irvine Machine Learning Repository (Housing, Machine CPU, Wine Quality, Automobile and Parkinson) and the 3 Use Cases (Protein Corona, Gajewicz Metal Oxides and Aquatic Toxicity) [file peerj-04-2721-s001.zip › 6_UseCases/3_AquaticToxicity/PLS.details.csv.repeatedcv.split1.pdf]

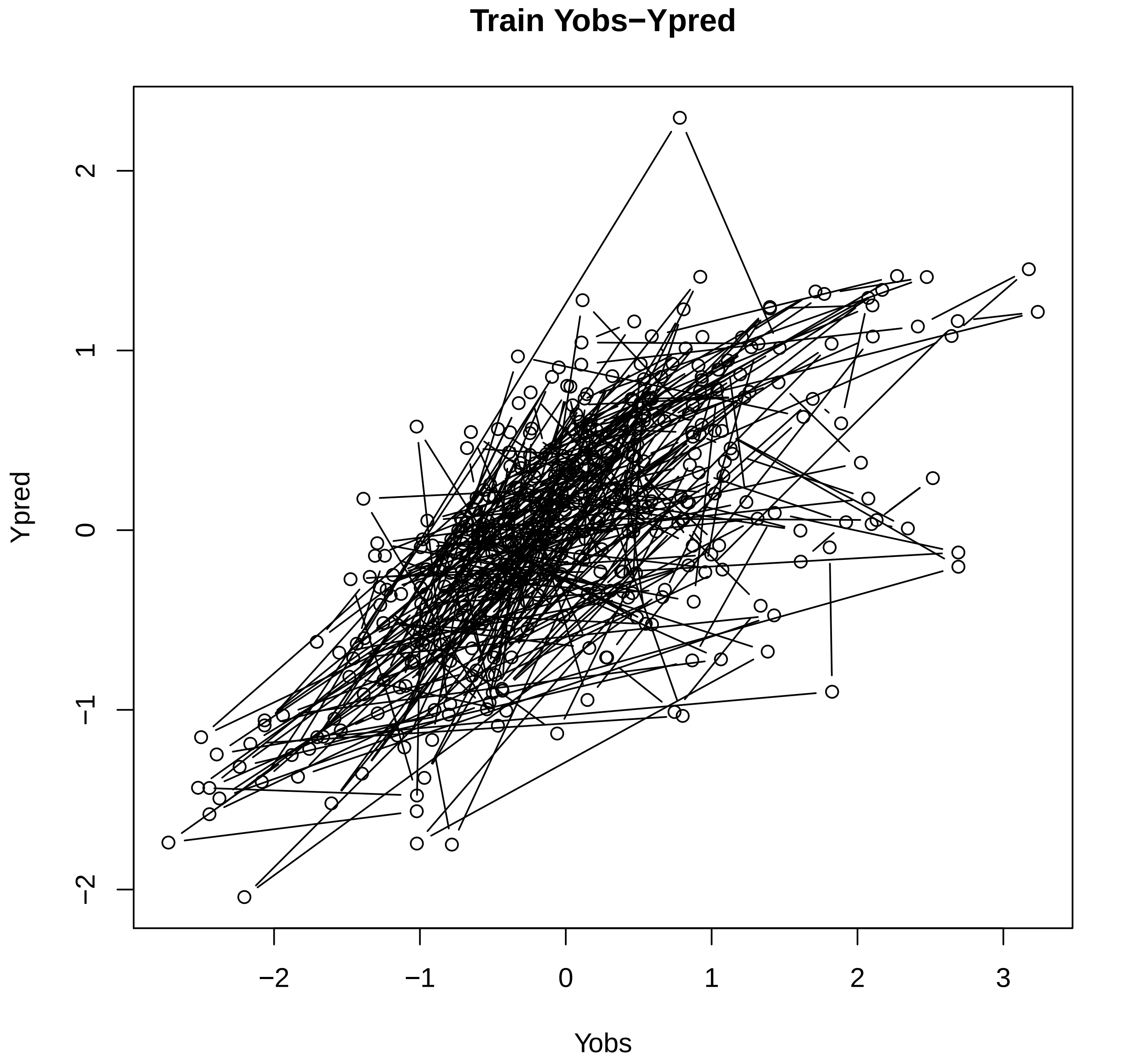

Train Yobs−Ypred
Ypred
Yobs
−2
−1
0
1
2
3

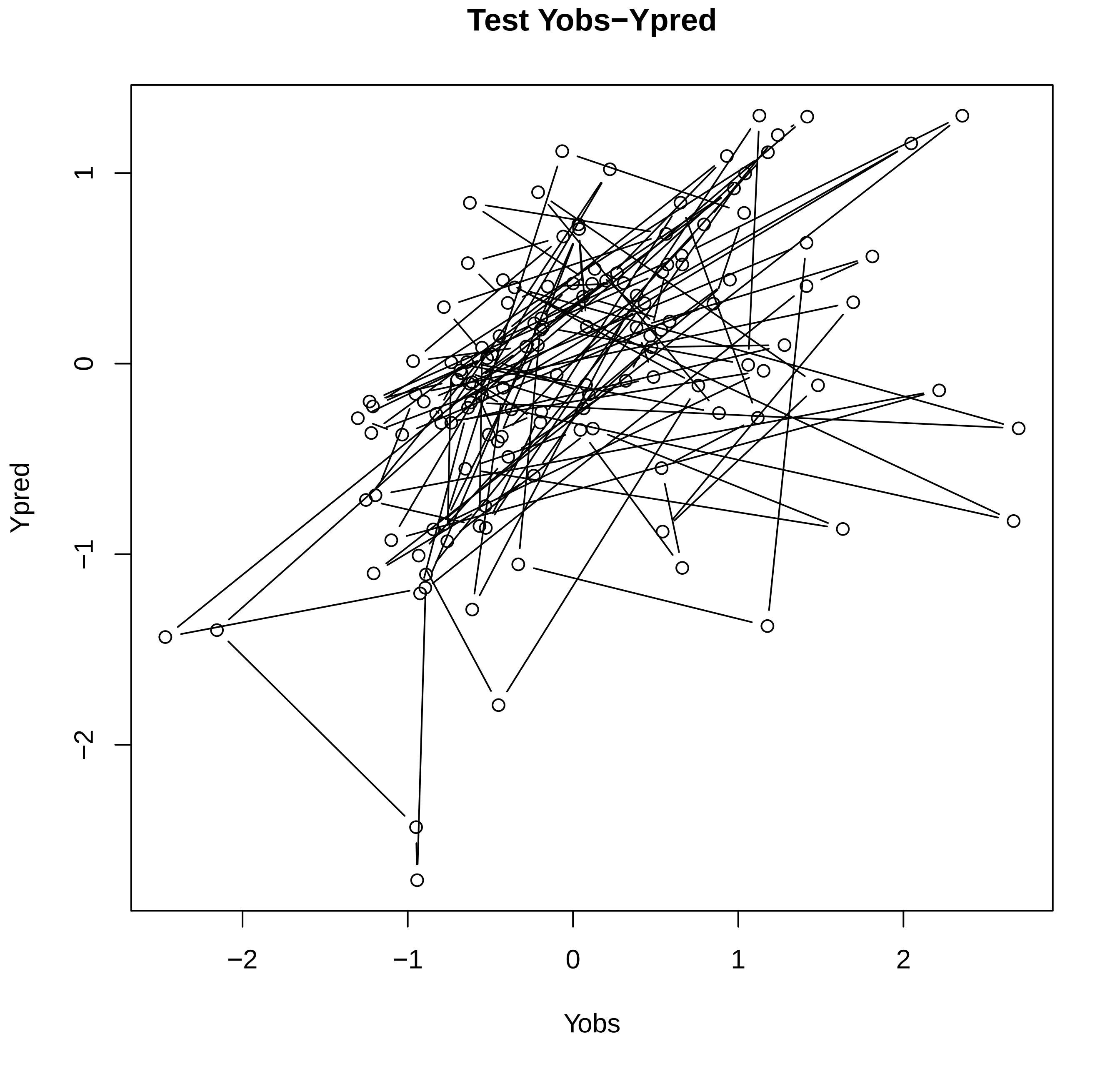

Test Yobs−Ypred
Ypred
1
0
−1
−2
−2
−1
0
1
2
Yobs

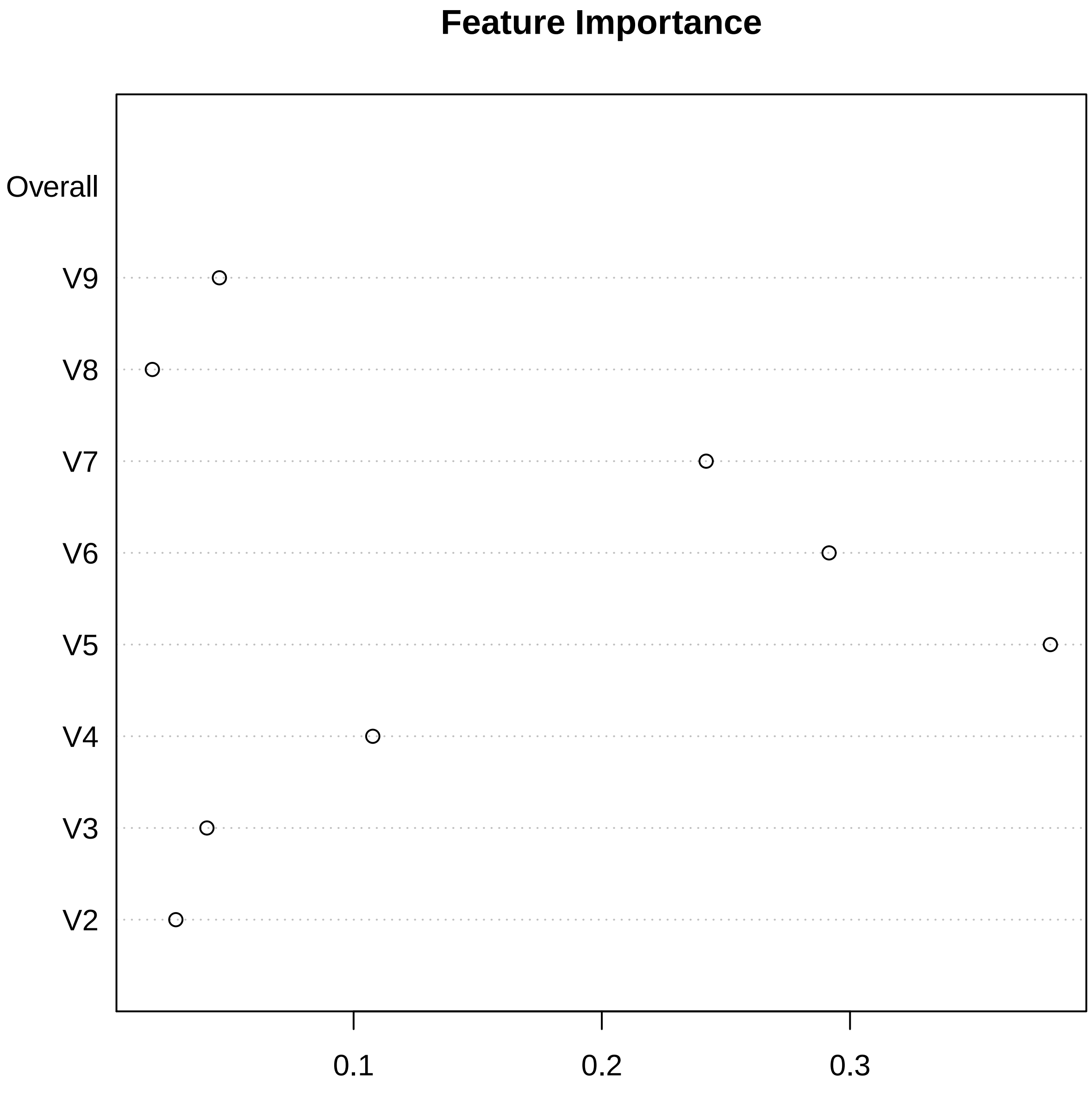
Feature Importance
Overall
V9
V8
V7
V6
V5
V4
V3
V2
0.1
0.2
0.3

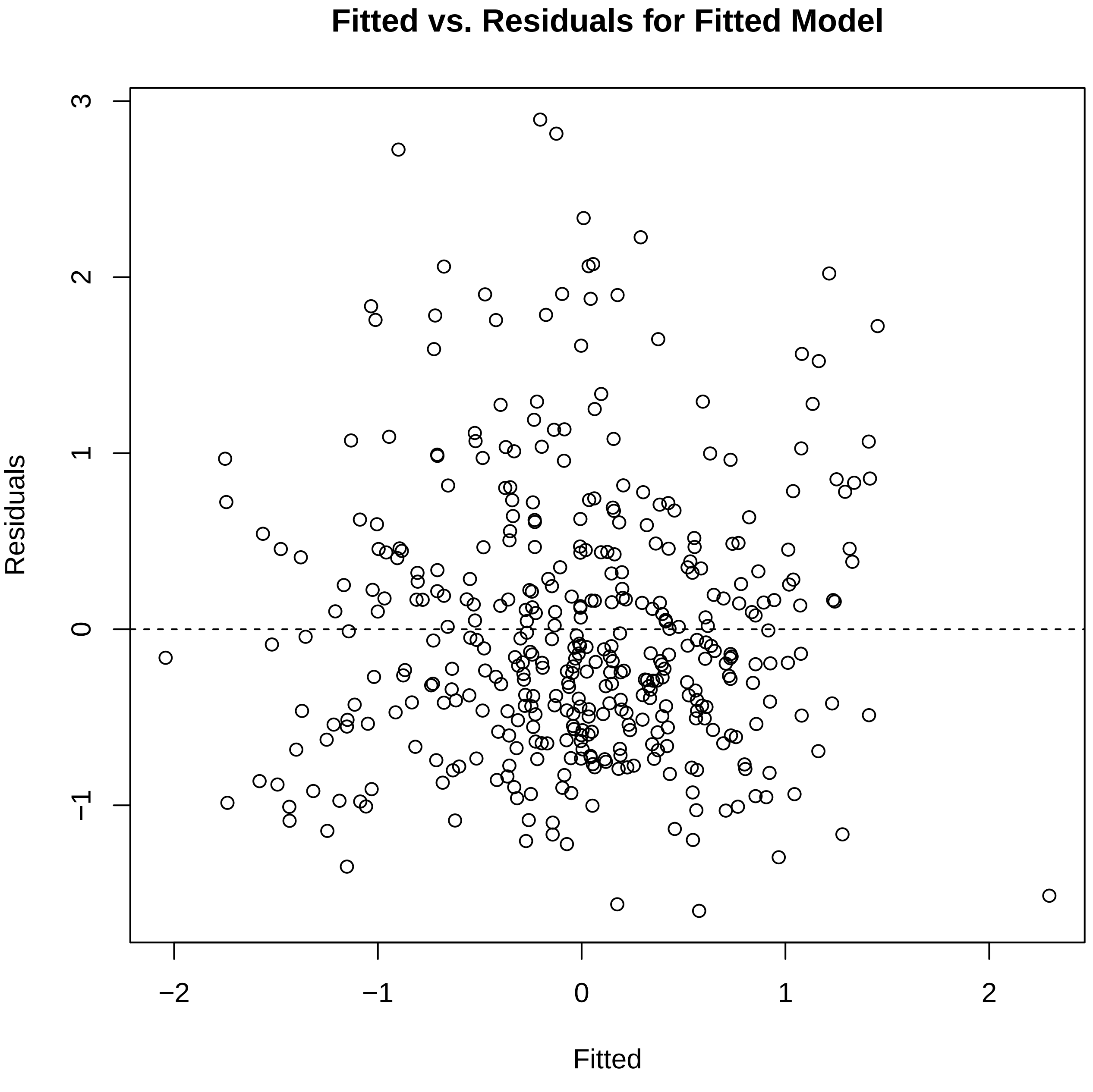

Fitted vs. Residuals for Fitted Model
Residuals
Fitted
−2
−1
0
1
2
3

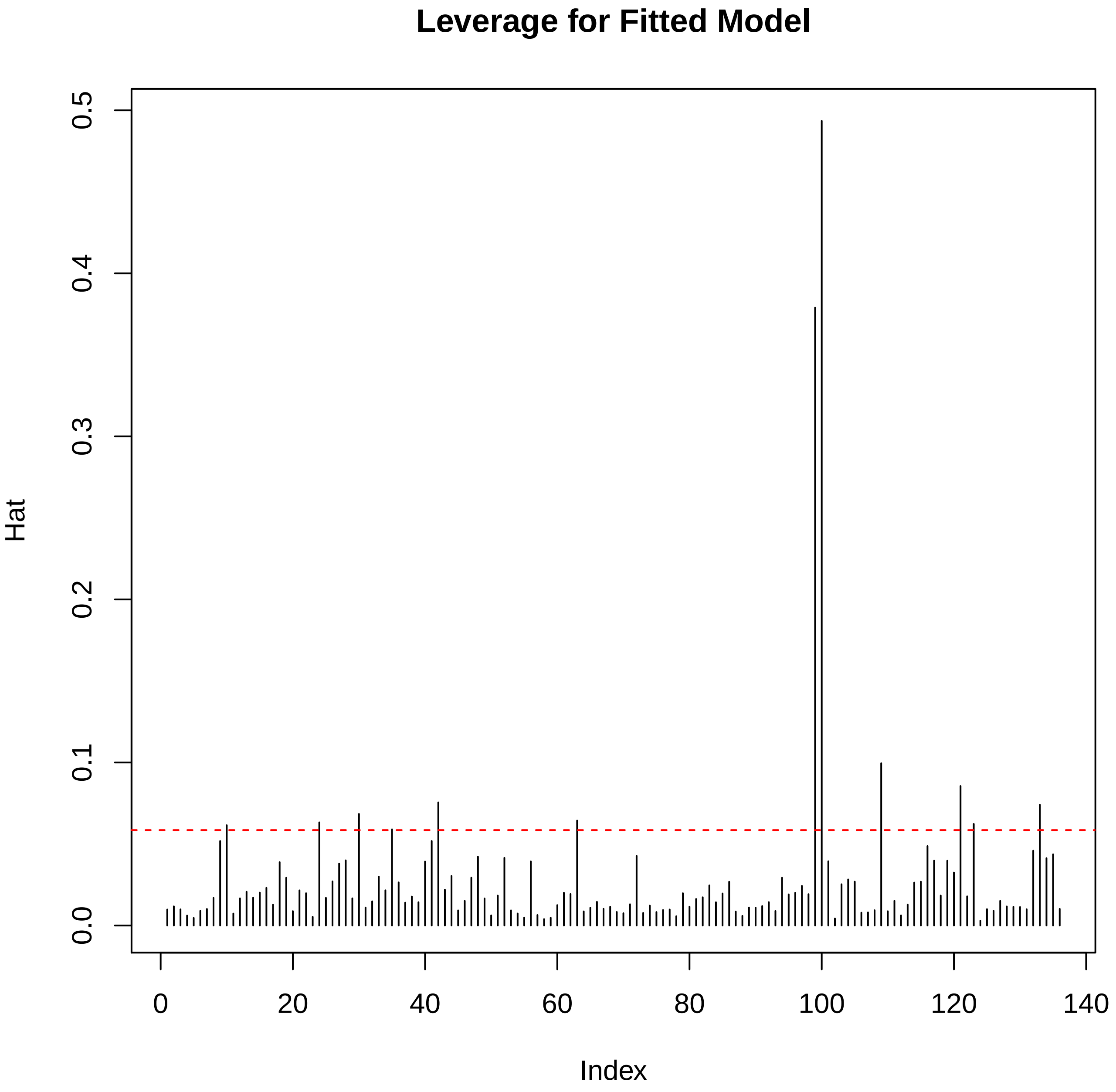

Leverage for Fitted Model
Hat
0.0
0.1
0.2
0.3
0.4
0.5
Index
0
20
40
60
80
100
120
140
